# 33.1 DRIVERS OF STIGMA FOR THE CLINICAL HIGH-RISK STATE FOR PSYCHOSIS—IS STIGMA DUE TO SYMPTOMS OR THE AT-RISK IDENTIFICATION ITSELF?

**DOI:** 10.1093/schbul/sby014.137

**Published:** 2018-04-01

**Authors:** Lawrence Yang, Bruce Link, Kristen Woodberry, Cheryl Corcoran, Caitlin Bryant, Donna Downing, Dan Shapiro, Francesca Crump, Debbie Huang, Drew Blasco, William McFarlane, Larry Seidman

**Affiliations:** 1 Columbia University Medical Center; 2 University of California at Riverside; 3 Beth Israel Deaconess, Harvard Medical Center; 4 Icahn School of Medicine at Mount Sinai; 5 University of Massachusetts/ Boston; 6 Maine Medical Center; 7 Columbia University; 8 New York University

## Abstract

**Background:**

The clinical high-risk state for psychosis syndrome (CHR) offers substantial potential benefits in terms of early identification and treatment for at-risk youth. Early treatment might lead to decreased symptoms, thus leading to reduced symptom-related stigma. However, stigma of the clinical high-risk state for psychosis designation might also initiate further stigma through the label of risk for psychosis. Identifying the effects of these sources of stigma is critical in order to best minimize stigma associated with CHR identification and to facilitate recovery.

**Methods:**

Baseline stigma assessments were conducted with 170 clinical high risk state for psychosis individuals in a major, NIH-funded longitudinal study at Columbia University Medical Center, Harvard University Medical Center, and Maine Medical Center from 2012 to 2017. Labeling-related measures of stigma (e.g., “shame of being identified as at psychosis-risk”) adapted to the CHR group, and a parallel measure of symptom-related stigma (e.g., “shame of the symptoms associated with CHR”) were administered. These measures were examined in relation to outcomes of: a) self-esteem, b) quality of life, and c) social functioning, adjusting for sociodemographics and core CHR symptoms (e.g. attenuated psychotic symptoms).

**Results:**

Results indicated that stigma related to symptoms was more strongly associated with all outcomes when compared with shame related to the risk-label. Stigma related to symptoms remained a significant predictor of self-esteem and quality of life even after accounting for stigma related to the risk-label and the effects of sociodemographics and CHR symptoms. Conversely, stigma related to the risk-label was no longer a significant predictor for outcomes after accounting for stigma related to symptoms.

**Discussion:**

Overall, symptom-related stigma was a more salient correlate and was independently linked with self-esteem and quality of life even after accounting for the effects of stigma related to the risk-label. These results indicate that treating of symptoms through early identification and treatment may provide major benefit for CHR youth by also alleviating symptom-related stigma. These findings also indicate that CHR services should address stigma associated with symptoms immediately at first identification, as these have substantial effects on psychological and functional outcomes. These findings have implications for guiding implementation of specialized CHR services both in the United States and worldwide.

